# From Non-symbolic to Symbolic Proportions and Back: A Cuisenaire Rod Proportional Reasoning Intervention Enhances Continuous Proportional Reasoning Skills

**DOI:** 10.3389/fpsyg.2021.633077

**Published:** 2021-05-21

**Authors:** Roberto A. Abreu-Mendoza, Linsah Coulanges, Kendell Ali, Arthur B. Powell, Miriam Rosenberg-Lee

**Affiliations:** ^1^Department of Psychology, Rutgers University, Newark, NJ, United States; ^2^Graduate School of Education, Rutgers, The State University of New Jersey, New Brunswick, NJ, United States; ^3^Department of Urban Education, Rutgers University, Newark, NJ, United States; ^4^Center for Molecular and Behavioral Neuroscience, Rutgers, The State University of New Jersey, Newark, NJ, United States

**Keywords:** proportional reasoning, intervention, non-symbolic processing, symbolic processing, inhibitory control

## Abstract

The persistent educational challenges that fractions pose call for developing novel instructional methods to better prepare students for fraction learning. Here, we examined the effects of a 24-session, Cuisenaire rod intervention on a building block for symbolic fraction knowledge, continuous and discrete non-symbolic proportional reasoning, in children who have yet to receive fraction instruction. Participants were 34 second-graders who attended the intervention (intervention group) and 15 children who did not participate in any sessions (control group). As attendance at the intervention sessions was irregular (median = 15.6 sessions, range = 1–24), we specifically examined the effect of the number of sessions completed on their non-symbolic proportional reasoning. Our results showed that children who attended a larger number of sessions increased their ability to compare non-symbolic continuous proportions. However, contrary to our expectations, they also decreased their ability to compare misleading discretized proportions. In contrast, children in the Control group did not show any change in their performance. These results provide further evidence on the malleability of non-symbolic continuous proportional reasoning and highlight the rigidity of counting knowledge interference on discrete proportional reasoning.

## Introduction

Learning fractions is an arduous and protracted process for students. In the United States, fraction instruction typically starts in third grade with fraction expressions; then, in fourth grade, children are first introduced to arithmetic operations with fractions (Common Core State Standards Initiative, 2020b). However, even after four years of instruction, less than a third of eighth-graders (∼30%) show an understanding of fraction addition (Carpenter et al., 1980; Lortie-Forgues et al., 2015). Regrettably, fraction arithmetic is just one example of students’ persistent difficulties with fractions (Siegler and Lortie-Forgues, 2017; van Hoof et al., 2018). Recent efforts from researchers and educators to develop novel methods involving non-symbolic representations to teach fractions are beginning to bear fruit. In the current study, we examined the effects of an intervention using Cuisenaire rods to improve non-symbolic proportional reasoning, a building block for symbolic fraction knowledge.

### Part-Whole and Alternative Models of Fractions

Traditionally, fractions are represented using part-whole models (e.g., pie charts). However, these representations might impede understanding of fundamental fraction properties, such as ratio, by promoting whole-number strategies, like counting (Plummer et al., 2017). In contrast, interventions that use non-symbolic continuous models, like number lines, provide a shared representation for whole and rational numbers, take advantage of spatial-numeric associations and capture the continuous property of fractions (Hamdan and Gunderson, 2017). These interventions also leverage children’s early proficiency at comparing and matching continuous proportional information (Boyer et al., 2008; Boyer and Levine, 2015; Hurst and Cordes, 2018). Indeed, one of the most promising methods to improve fraction skills is intensive training involving mapping non-symbolic continuous representations of proportions with fractions (Fazio et al., 2016; Braithwaite and Siegler, 2020; Soni and Okamoto, 2020; Wortha et al., 2020).

Emerging evidence from individual difference studies and experimental research also supports the link between non-symbolic continuous representations of proportions and fraction skills (Matthews et al., 2016; Bhatia et al., 2020; Kalra et al., 2020). For instance, college students who are more precise in their judgments of non-symbolic ratios are also better at comparing symbolic fractions (Matthews et al., 2016). Moreover, matching non-symbolic continuous representations of proportions to symbolic fractions is modulated by the distance effect (e.g., lower performance in comparing smaller ratios than larger ratios), suggesting that both formats activate the same mental proportional magnitude representations (Bhatia et al., 2020). Overall, these findings indicate that students might use their non-symbolic continuous proportional reasoning skills as a scaffold for symbolic fraction knowledge. Yet, little is known about the malleability of non-symbolic proportional reasoning through training.

### Training Non-symbolic Proportional Reasoning

#### Continuous Non-symbolic Proportions

To date, only two studies have reported changes in non-symbolic continuous proportional reasoning following training. These studies employed individual, computerized interventions with carefully matched control conditions (Gouet et al., 2020; Wortha et al., 2020). In Gouet et al. (2020), nine-year-old children went through one of two non-symbolic continuous proportional interventions or an absolute magnitude control condition. In both non-symbolic interventions, children used a number line to estimate proportional continuous quantities, either the red area of a two-color rectangle or the size of a yellow circle relative to a blue circle. After the five-day intervention, children from both interventions improved their non-symbolic proportional skills, while children in the control group, who only practiced absolute magnitude comparison skills, did not. Crucially, the intervention also had a positive effect on children’s symbolic fraction arithmetic and comparison skills.

In the second study, Wortha et al. (2020) examined the effects of fraction intervention on adults’ reaction times and brain activation while performing three tasks: a cross-format proportional matching task, a number line comparison task, and a fraction comparison task. Their results showed that after estimating fraction magnitudes using number lines for five days, participants became more precise in matching number lines to fractions and comparing number lines after the intervention. However, they showed no gains in their symbolic fraction comparison skills. The brain imaging results showed the opposite pattern: there were no changes in the activation during the matching and number line comparison tasks, but during the symbolic fraction comparison task, activation increased in a set of frontoparietal regions implicated in math cognition, including the bilateral intra-parietal sulcus and the middle and the inferior gyrus. Together, these studies suggest that non-symbolic continuous proportional reasoning can be improved by exclusively training non-symbolic skills or by matching symbolic and non-symbolic proportions.

**FIGURE 1:**
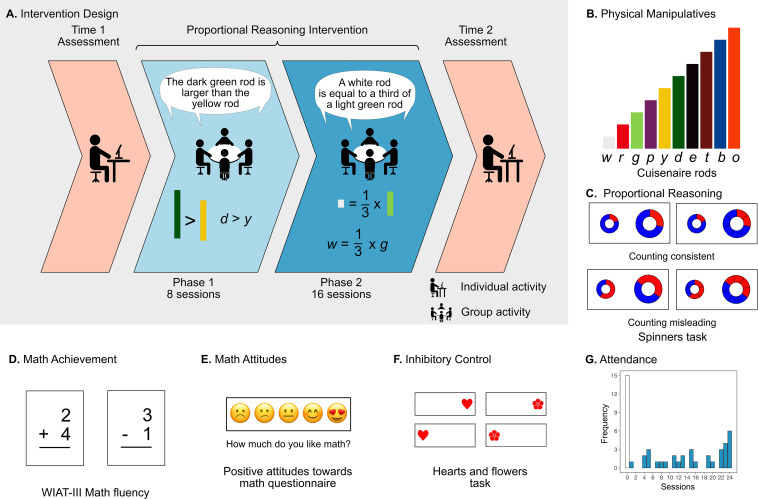
Study design. **(A)** The study consisted of three stages: the time 1 assessment (late September 2018), 24 sessions of a group-based proportional reasoning intervention, and (3) a time 2 assessment (March 2019), which repeated the time 1 assessment. The intervention was further divided into two phases: Phase 1 (8 sessions), in which children became familiarized with the materials and manipulated absolute magnitudes, physically, orally and symbolically; Phase 2 (16 sessions), in which children represented and compared non-symbolic and symbolic proportions. **(B)** During both phases, children initially worked with the physical rods and then they used letters to refer to the rods (w = white, r = red, g = green, p = purple, y = yellow, d = dark green, e = ebony, t = tan, b = blue, o = orange). The time 1 and time 2 assessment comprised of **(C)** the spinners task, the main outcome task, which comprised continuous (left pairs spinners) and discrete (right pairs of spinners) formats **(D)** the arithmetic fluency subtests from the Wechsler Individual Achievement Test (WIAT)-III, **(E)** the positive attitudes toward math questionnaire, and **(F)** the hearts and flowers task. **(G)** The histogram shows the number of children who attended a given number of sessions, from zero sessions (control group) to 24 sessions (complete intervention).

These studies employed strictly controlled computerized interventions, which lack ecological validity, and their implementation in traditional classrooms might be technologically challenging. In contrast, for the current study, we implemented a proportional reasoning intervention in classrooms and with inexpensive physical manipulatives. Using well-known educational materials, Cuisenaire rods, children transitioned from comparing the relative lengths of pairs of rods to expressing those comparisons symbolically (Figures 1A,B). Thus, our first aim was to examine the possible positive transfer effects of this intervention on the ability to compare proportions presented in another non-symbolic format (i.e., annulus-shaped figures, Figure 1C) in second-grade children who have yet to receive formal fraction instruction.

#### Discrete Non-symbolic Proportions

The ease with which children can represent non-symbolic continuous proportions contrasts with the great difficulty they encounter when comparing discrete proportions (Jeong et al., 2007; Boyer et al., 2008; Hurst and Cordes, 2018). For instance, while even four-year-old children can successfully compare non-symbolic continuous proportions, ten-year-old children still struggle with discrete ones, particularly when the discrete information contradicts proportional information (Jeong et al., 2007). Children’s tendency to exert whole-number strategies to proportional reasoning tasks is consistent with similar phenomena seen in symbolic fraction comparison, an effect termed whole number bias (Ni and Zhou, 2005). Given the persistent developmental challenge entailed by discrete proportional reasoning, an outstanding question is whether interventions that improve continuous proportional reasoning influence discrete proportional reasoning skills. The second aim of this study is to shed light on this question.

A small set of studies have investigated how non-symbolic proportional comparison skills relate across formats (Mock et al., 2018; Park et al., 2020). Recently, Park et al. (2020) examined non-symbolic proportional comparison skills in preschoolers, second graders, fifth graders, and adults across continuous (circles, lines, and blob areas) and discrete (collections of circles) non-symbolic formats. The authors also evaluated absolute magnitude comparison skills across these four formats. They report that proportional skills in one format were better predicted by proportional skills in another format than absolute magnitude comparison skills of the same format. For instance, comparing the ratio between pairs of circles was better predicted by comparing the ratio between pairs of lines than comparing the absolute magnitude of circles. These results suggest that individuals use the same proportional comparison capacity regardless of the format in which proportions are presented. Consistent with these results, Mock et al. (2018) found in adults, overlapping brain regions for the processing of proportions presented as non-symbolic continuous and discrete representations, fractions, and decimals, as do other rational numbers (Rosenberg-Lee, 2021). These regions were the superior parietal lobule, the inferior, middle, and superior occipital gyri. Together, these studies suggest that improvements in one format should also be reflected in other formats due to proportional magnitudes being processed in an amodal manner. However, this conclusion is difficult to reconcile with the persistent decrements in performance found in non-symbolic discrete formats (Jeong et al., 2007; Begolli et al., 2020). An alternative line of research (Boyer and Levine, 2015; Hurst and Cordes, 2018; Abreu-Mendoza et al., 2020) suggests priming continuous proportional reasoning immediately before discrete stimuli mitigates the challenges of discrete proportional reasoning. The current study aimed to examine whether an intervention focused on non-symbolic continuous skills positively shapes children’s non-symbolic discrete proportional reasoning skills.

### Domain-Specific and Domain-General Predictors of Intervention Effects

The current intervention design also allowed us to examine the predictor effects of mathematical achievement and attitudes, as well as cognitive skills that previous intervention studies have shown play a critical role in fraction learning.

#### Math Abilities

Only a few studies have examined the moderating role of student’s general math knowledge on fraction learning. In one study, Fuchs et al. (2013) showed that children’s initial scores in the National Assessment of Educational Progress assessment did not influence children’s gains from a 12-week fraction intervention. Conversely, Reinhold et al. (2020) found that prior math achievement, measured by the type of school (high and low achieving institutions), moderated the effects of different types of fraction interventions. High-achieving children showed larger gains from a new fraction curriculum than a traditional one, regardless of whether it was presented as either a book or an e-book. However, low-achieving children only benefited from the new curriculum when it was offered as an e-book. Longitudinal studies of fraction learning have shown that initial general math performance is a predictor for later conceptual and procedural fraction knowledge (Jordan et al., 2013). Together, these studies suggest the potential for a positive relationship between general math knowledge and fraction instruction; however, conclusive evidence is still emerging.

#### Attitudes Toward Math

Holding more positive attitudes toward mathematics is positively related to math performance (Chen et al., 2018; Dowker et al., 2019). Yet, little is known about the specific relations of math attitudes to non-symbolic and symbolic proportional reasoning. Recently, Sidney et al. (2019) found that children and adults had more negative attitudes toward fractions than whole numbers. Further, while children’s attitudes toward whole-numbers and fractions were equally related to general math performance, adults’ attitudes toward whole-numbers were more strongly associated with math than attitudes toward fractions. In the current study, we investigated whether positive attitudes toward math, in general, are predictive of learning gains in non-symbolic proportional reasoning.

#### Executive Functions and Inhibitory Control

Inhibitory control plays a critical role in acquiring information that contradicts previously learned knowledge both in science and math (Brookman-Byrne et al., 2018). Specific to fraction learning, inhibitory control may help students override automatic whole-number representations and hone in proportional magnitudes (Vosniadou et al., 2018). Studies of individual-differences finds that students with higher inhibition capacity are better at comparing misleading non-symbolic discrete proportions (Abreu-Mendoza et al., 2020), misleading fractions (Gomez et al., 2015; Avgerinou and Tolmie, 2019), and misleading decimals (Avgerinou and Tolmie, 2019; Coulanges et al., 2021). Here, we aimed to extend these findings by examining the predictive role of inhibitory control in learning gains in non-symbolic proportional reasoning, specifically when there is a need to disregard misleading discrete information.

Although these individual-difference studies suggest a positive relationship between learning gains and inhibitory control, a previous study of the moderator effect of working memory, another canonical executive function (Diamond, 2013), on fraction interventions alludes to a more nuanced relationship (Fuchs et al., 2014). In their study, the contribution of working memory varied depending on the type of intervention. While participants with low working memory benefitted most from a conceptually rich fraction intervention, participants with high working memory levels showed the largest gains when the intervention focused on fraction arithmetic fluency. Overall, these findings indicate that inhibitory control may play a key role in improving non-symbolic proportional reasoning, but its effects could depend on the kind of instruction.

### The Current Study

The aims of this study were threefold: (1) provide further evidence on the malleability of non-symbolic continuous proportional reasoning in the context of a classroom-based, physical manipulatives intervention; (2) investigate whether an intervention that targets continuous representations of fractions leads to improvements in discrete proportional reasoning; (3) examine possible academic, attitudinal, and cognitive predictors of children’s improvement in non-symbolic proportional reasoning. To achieve these study aims, second graders participated in a 24-session intervention program that introduced fractions as multiplicative comparisons between two continuous quantities. Specifically, students measured the length of rods of different sizes and learned to communicate in oral and written forms the relationship between the rod lengths.

Consistent with previous findings, we predicted that children’s non-symbolic continuous proportional reasoning would increase following the intervention. We further hypothesized that discrete comparison skills, particularly in contexts where discrete information is misleading, would also improve. Based on the finding that inhibitory control relates to misleading discrete proportional reasoning, we tested the hypothesis that children with high inhibition skills would show larger learning gains in discrete misleading trials. These two hypotheses were pre-registered on a pre-registration poster submission (Mathematical Cognition and Learning Society, 2019). Finally, we predict that children with strong initial math skills and more positive attitudes toward math will show larger proportional reasoning gains than those with low math skills and less favorable attitudes.

## Materials and Methods

### Participants and Intervention Phases

Fifty-seven students from three second-grade classrooms at a public school in Newark, NJ, were invited to participate in this study. Of the 52 children whose parents consented for them to participate in the study, 50 participants completed the two assessment sessions. Out of these students, 35 children were part of an after-school enrichment program that comprised the original intervention group. Among these 35 children, the final sample excluded one participant because they did not have the minimum number of usable trials in the key outcome measure (see Section “Proportional Reasoning”). Thus, the final sample of the intervention group consisted of 34 children. However, as attendance at the intervention sessions was irregular (median = 15.6 sessions, range = 1–24, Figure 1G), we used the number of sessions completed as an independent variable for all our analyses. Children who did not participate in the after-school program (*n* = 15) comprised the original control group, and they were coded as having 0 sessions in any analyses which used attendance as a continuous variable. All children’s parents gave written informed consent, and the children gave oral assent for their participation. The Rutgers University Institutional Review Board approved the research protocol. The pre-training data from this sample is reported elsewhere (Abreu-Mendoza et al., 2020).

We performed a sensitivity power analysis using the final sample size (*n* = 49) as a reference for the planned correlations. These analyses indicated that our sample size enables detecting moderate to large correlations (Pearson’s *r* > 0.38) using an alpha = 0.05 and power = 0.80. Using the same alpha and power values, in the intervention group, we can detect medium to large correlations (Pearson’s *r* > 0.44) while in the control group only large effects (Pearson’s *r* > 0.62).

### Study Overview

The study consisted of three stages: (1) a pre-intervention assessment (time 1, late September 2018), which consisted of four activities, administered in the following order: Math fluency subtests (math achievement), Spinners task (proportional reasoning), Hearts and Flowers (H&F, inhibitory control) task, and Positive Attitudes toward Math questionnaire; (2) 24 sessions of a group-based proportional reasoning intervention; and (3) a post-intervention assessment (time 2, March 2019), in which participants performed the same activities as the pre-intervention assessment, in the same order (Figure 1). Children in the control group only completed stages 1 and 3.

The two computerized tasks, the Spinners and H&F tasks, were presented using PsychoPy2 Experiment Builder Version 1.90.3 (Pierce, 2007). Children were evaluated individually by trained experimenters in quiet corners of a large room at the children’s school (maximum of 4 children at a time). Experimenters were blind to the group assignment of participants. Each assessment session lasted approximately 25 min.

### Academic, Cognitive, and Attitude Assessments

#### Mathematical Achievement

As fractions are not typically taught in second grade in the United States (Common Core State Standards Initiative, 2020b), to evaluate children’s mathematical achievement, we concentrated on skills appropriate for children’s academic stage (i.e., arithmetic skills). Thus, we used the Math Fluency–Addition and Math Fluency–Subtraction subtests from the Wechsler Individual Achievement Test–Third Edition (WIAT- III; Wechsler, 2009). In each subtest, children answered as many arithmetic (first addition, then subtraction) problems as they could in 1 min. Combining the grade-normed scores of each subtest provides an age-appropriate measure of children’s mathematical achievement.

#### Attitudes Toward Mathematics

To evaluate children’s attitudes toward math, we adapted the 5-point Likert-type Positive Attitude toward Math (PAM) questionnaire (Chen et al., 2018). To make it appropriate for children, we used emojis to help connote the response options. This questionnaire was comprised of six items that evaluate children’s attitudes toward math and six items that evaluate their general attitude toward academics (e.g., science, reading, computers, and technology). For this study, our variable of interest was the average of the first six questions relating to math attitudes.

#### Inhibitory Control

Children’s inhibitory control was assessed with the Hearts and Flowers task (hereafter H&F task; Davidson et al., 2006; Brocki and Tillman, 2014; Wright and Diamond, 2014). This computerized task consisted of three blocks presented in the following fixed order: congruent, incongruent, and mixed. The experimenter read aloud the on-screen instructions to the children. In the congruent block, children were instructed to press the key on the same side as where the target (hearts) appeared, using the keys “z” for the left side and “m” for the right side. In the incongruent block, children were instructed to press the key on the opposite side from where the target (flowers) appeared. In the mixed block, children saw interspersed hearts and flowers and were asked to respond according to the previously learned rules. At the beginning of the congruent and incongruent blocks, there were 2 example trials. The corresponding figure (heart or flower) appeared first on the right and then on the left. The target images remained on the screen until the children pressed the correct key. The first two blocks comprised 12 trials each, which randomly presented the corresponding figure on each side six times. The third (mixed) block contained 33 trials, and the first trial of this block was always a heart presented on the right side. Subsequent trials randomly presented each figure 16 times, eight times on each side. We considered this last block as the measure of inhibitory control because prior research found that performance in the mixed block was strongly correlated with a latent variable of inhibition (*r* = 0.71), whereas performance in the congruent block and performance in the incongruent block were negative (*r* = −0.03) and weakly associated (*r* = 0.17), respectively (Brocki and Tillman, 2014).

Following Wright and Diamond (2014), when computing accuracy, we excluded anticipatory responses (reaction times [RT] shorter than 250 ms) and outlier responses (RTs at least 3 standard deviations above the individual’s mean). After applying these criteria, among the 49 children, we analyzed 4397 (96.61%) of 5586 trials.

### Outcome Task

#### Proportional Reasoning

To measure children’s learning gains in proportional reasoning, we used a computerized version of the Spinners task (Jeong et al., 2007). In this task, children saw two spinners and had to indicate which of them has a proportionally larger red area.

The 12 proportions used by Jeong et al. (2007) were presented in three different format blocks for a total of 36 experimental trials. In the continuous format, each spinner had only two continuous sections, one red and one blue. In the discrete adjacent format, the two continuous parts were segmented into discrete but adjacent sections of red and blue sections. In the discrete mixed format, the red and blue segments were interspersed. In the discrete blocks, the number of segments was manipulated so that in half of the trials, the spinner with the larger number of red pieces was also the one with the proportionally larger red area (counting consistent trials). In contrast, in the other half, the spinner with the fewer red pieces was the one with the proportionally larger red area (counting misleading trials). Although “counting” information could not be meaningfully assessed in the continuous format, trials that had the same proportions as the counting consistent trials of the discrete formats were considered continuous “counting consistent” trials by convention. Similarly, continuous trials that showed the same proportions as the counting misleading trials were considered continuous “counting misleading” trials.

For all formats, we also manipulated the size of the individual spinners to prevent children from relying exclusively on the red area’s absolute size in making their selections. Thus, on half of the trials of each format, the physically larger spinner also had the proportionally larger red area (size congruent trials). On the other half, the opposite pattern held, with the smaller spinner being the one with the proportionally larger red area (size incongruent trials). Spinners could be 6, 9, or 12 cm in diameter. For size congruent trials, the proportionally larger spinner was always the 12-cm spinner, and the other spinner could be 6 or 9 cm. In contrast, for size incongruent trials, the proportionally larger spinner was the 6-cm spinner. The other spinner was 9 or 12 cm. Proportion pairs (size congruent or size incongruent) were counterbalanced across participants. For all participants, the continuous condition was presented first, and the presentation order of the two discrete blocks was counterbalanced across participants. Importantly, children saw the same order in both time 1 and time 2 sessions.

For all conditions, trials started with a blank screen presented for 500 ms, followed by the pair of spinners. Spinners remained on the screen until the children responded by pressing one of two possible keys, “z” for the left spinner or “m” for the right one. Within each block, half of the correct responses were presented on the left and the other half on the right. More details about this task can be found at Abreu-Mendoza et al. (2020).

For consistency with the inhibitory control measure, we followed the same procedures for the H&F task when computing accuracy, which involved removing anticipatory and outlier responses. After applying these criteria, one participant who completed the intervention sessions did not have at least one trial from each type and was excluded from the final sample. Among the 49 children of the final sample, we analyzed 3429 (97.19%) of 3528 trials. For each participant, trial-level accuracy on this task was initially averaged by size (2), counting (2), format (3), and time (2), producing 24 data points per participant. However, given the complexity of the design and our theoretical interest in counting interference, representational format, and change over time, we then averaged across size, reducing the number of data points to 12 per participant. This approach provides a better estimate of performance within each trial type (e.g., counting misleading discrete adjacent at time 1) when there are unequal numbers of size congruent and incongruent trials (Abreu-Mendoza et al., 2020).

### Intervention Program

The group-based proportional reasoning intervention program consisted of 24 one-hour sessions, which children attended twice a week. Throughout the sessions, students transitioned from representing proportions using manipulatives (i.e., Cuisenaire rods) to writing fraction expressions symbolically.

The intervention was divided into two phases: In Phase 1, children were introduced to the Cuisenaire rods, agreed on names for each different color rod, and a single letter to represent each rod color (usually the initial letter of the color name, see Figure 1B). This phase involved activities in which children internalized the correspondence between the rods’ length and their colors and the relations of equality, inequality, and transitivity among the rods’ lengths. For example, one activity involved asking children to close their eyes while a peer placed a rod in their hands and asked them to say aloud the color of the rod they are holding. Children were also asked to compare the different sizes of the rods relative to others (e.g., “the yellow rod is larger than the green rod”) and place end-to-end, creating trains of rods of different sizes to equal the length of a larger rod. Then, children discussed the rods’ lengths without having the rods present and verbally discussed relations among rod lengths and trains of rods. Later, using the letters to refer to rods, they wrote symbolic expressions such as “*y* is larger than *g*” or “*yg*.” These tasks allowed children to move from non-symbolic to symbolic representations of lengths and relations among rod lengths. By the end of this phase, they could mentally evoke images of absolute magnitudes among rods of different lengths and symbolically represent those relations.

Phase 2 had four modules, which focused on the relative magnitude of the rods: In the first module, children were taught to use the rods as a tool to measure the length of other rods, which led to fractional expressions (e.g., “a white rod equals a third of a green rod”). Children described these relationships verbally with and then without the rods. In the second module, children used only symbols (i.e., operation and equality signs and letters) to refer to the rods and to write fractional expressions; for example, they might have written “*w* = 1/3×*g*.” Children were then taught how to compare the proportional quantities of these expressions, which led to the final two modules. In the third module, children replaced letters with expressions that referred to the same magnitude; instead of writing *w*, they wrote expressions like the following “1/3 × *g*.” In the final module, children compared these symbolic fraction expressions (e.g., “1/3 × *g* < 5/7 × *e”*).

During the two phases of the intervention, there were moment-by-moment formative assessments given by the instructors; however, there were no traditional summative assessments. Sessions were carried out by members of the research team: a university professor and a doctoral student. Further details of the program are reported in Powell (2019).

### Statistical Analyses

Our first approach to examine training-related changes in the performance of children who participated in the intervention, was to contrast the performance of the control and the intervention groups across the three formats of the Spinner task, our outcome measure. Table 1 shows the means and standard deviations for each group (control and intervention) across formats (continuous, discrete adjacent, and discrete mixed), counting conditions (consistent and misleading), and time (T1 and T2). For these three ANOVAs, we included time and counting as within-participant factors and group as a between-participant factor. However, with one exception (a marginal three-way interaction between counting, time, and group (*p* = 0.096) in the continuous format), there were no time by group interaction to indicate greater learning in the original intervention group. Noting the irregular attendance of the intervention (1–24 sessions), we instead adopted a dose-response framework and performed retrospective analyses (Voils et al., 2014) on the effect of the number of sessions attended on changes in children’s performance. Similar approaches have been employed to analyze results of educational interventions with incomplete attendance (Roberts et al., 2018), finding that when attrition rates are high and attendance is irregular, intervention effects might be better characterized by the mediator effects of the number of completed sessions.

**TABLE 1:** Mean (*SD*) performance across each format of the Spinners task by group.

**Continuous format**				
	**Intervention (*n* = 34)**		**Control (*n* = 15)**	
	**T1**	**T2**	**T1**	**T2**

Consistent	0.64 (0.25)	0.79 (0.18)	0.73 (0.23)	0.68 (0.21)
Misleading	0.64 (0.28)	0.69 (0.25)	0.71 (0.21)	0.74 (0.18)

**Discrete adjacent format**				

Consistent	0.79 (0.21)	0.91 (0.14)	0.78 (0.26)	0.80 (0.25)
Misleading	0.58 (0.25)	0.50 (0.25)	0.50 (0.30)	0.51 (0.29)

**Discrete mixed format**				

Consistent	0.85 (0.21)	0.86 (0.22)	0.77 (0.25)	0.88 (0.21)
Misleading	0.50 (0.29)	0.43 (0.26)	0.42 (0.34)	0.46 (0.33)

Specifically, we computed linear mixed effects models using attendance as one of the between-participant fixed factors. To facilitate interpretation of results for all measures, we first standardized T1 and T2 scores relative to the T1 means and standard deviations. Therefore, all average performance at T1 measures would be centered around 0, and increments or decrements in the T2 session are reported in terms of standard deviations. Therefore, using the same guidelines as Cohen’s *d* the resulting beta values can be interpreted using the follow criteria *small* 0.2 to < 0.5, *medium* 0.5 to < 0.8, and *large* 0.8 and above. Finally, to further characterize the main effects and interaction, we obtained the marginalized means at four levels of attendance (0, 8, 16, 24 sessions). We then performed pairwise comparisons at each level to determine if fitted increments or decrements differed significantly from zero. All statistical analyses were conducted in R 3.5.3 (R Core Team, 2019), linear mixed models were computed using the *lme4* package (Bates et al., 2015) and pairwise comparisons were performed using the *lsmeans* package (Lenth and Lenth, 2018). To investigate the effects of predictor variables, we first used Pearson’s correlations between the learning gains in our outcome measure, the spinners task, across its different formats with the time 1 scores in the math, attitudinal, and inhibitory control measures. In cases where there was a significant correlation in the intervention group but not the control, we performed standard linear regressions with learning gains in the spinner task as the dependent variable, and examined interactions between number of sessions and the significant predictors to determine if the relations were specific to the training group. This approach also accounts for differences in sample size between the intervention and control group and hence the resulting power differences (Section “Participants and Intervention Phases”).

## Results

### Cognitive and Attitude Assessments

#### Pre-intervention Assessment

Table 2 shows the detailed descriptive statistics of the mathematical achievement, mathematical attitudes, and inhibitory control skills, before and after the intervention for both children who attended the intervention and children in the control group. Importantly, there were no differences in children’s age, mathematical achievement, attitudes toward math, nor inhibitory control across the two groups before the intervention. Further, using attendance as a continuous variable there were no correlations of attendance with these demographic, academic, attitudinal, and cognitive variables (absolute *rs* < 0.15, *ps* > 0.32). Overall, these results suggest that any observed relationships between attendance and changes in proportional reasoning are not due to prior differences in math achievement or attitudes, or cognitive differences.

**TABLE 2:** Group demographic, academic, and cognitive characteristics.

	**Intervention group (*n* = 34)**	**Control (*n* = 15)**	***t*(47)**	***p*-value**
Gender	15F/19M	6F/9M		
**Pre-training**				
Age	7.60 (0.38)	7.50 (0.27)	0.89	0.38
WIAT Math	81.12 (9.64)	80.80 (11.35)	0.10	0.92
ATM	4.05 (0.84)	4.29 (0.59)	1.00	0.32
H&F congruent	0.87 (0.12)	0.88 (0.14)	0.35	0.73
H&F incongruent	0.67 (0.31)	0.63 (0.28)	0.37	0.71
H&F mixed	0.55 (0.14)	0.50 (0.12)	1.33	0.19
**Post-training**				
Age	8.04 (0.37)	7.95 (0.26)	0.87	0.39
WIAT Math	88.18 (12.10)	88.00 (12.77)	0.05	0.96
ATM	4.24 (0.73)	4.15 (0.82)	0.38	0.70
H&F congruent	0.90 (0.12)	0.88 (0.13)	0.37	0.71
H&F incongruent	0.77 (0.26)	0.75 (0.24)	0.28	0.78
H&F mixed	0.59 (0.19)	0.54 (0.14)	1.05	0.30

*H&F, Hearts and Flowers task; ATM, Attitudes Toward Math.*

#### Time 1 to Time 2 Intervention Changes in Predictor Measures

**FIGURE 2:**
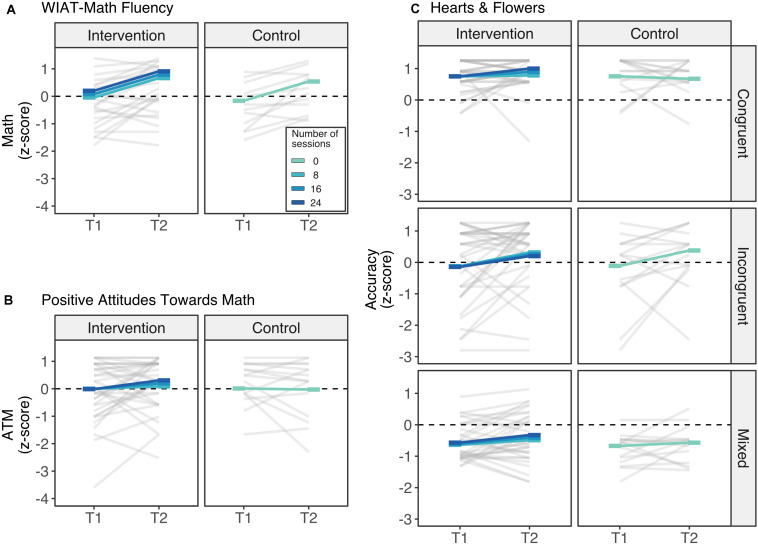
Time 1 (T1) to time 2 (T2) changes in z-scores of the **(A)** WIAT-III Math Fluency composite scores, **(B)** Positive attitudes toward math scores, and **(C)** Hearts and Flowers’ accuracy. Over the five months between time 1 and time 2 and regardless of the number of completed sessions of the intervention, children improved their math skills above and beyond what was expected during this period **(A)**. While children’s attitudes toward math remained stable from time 1 to time 2 **(B)**, children also improved their performance in the incongruent block of Hearts and Flowers tasks **(C)**. Rods represents the estimated marginal means across different number of sessions of attendance from 0 sessions (control group) to 24 sessions (darkest blue).

To examine changes in the predictor measures over the five months between time 1 and time 2 and to determine whether attendance to the intervention program modulated these changes, we performed three linear mixed repeated models, which included time (T1 and T2) as a within-participant fixed factor and attendance as a between-participant fixed factor, and participants as a random factor with the standardized scores of each assessment as the dependent measure (Figure 2). For the H&F task, we also used block as a within-participant fixed categorical factor (congruent, incongruent, and mixed).

**Mathematics achievement.** There was a main effect of time (beta = 0.702, *SE* = 0.180, *t*(47) = 3.91, *p* < 0.001) for the arithmetic fluency scores of the WIAT-III. As we used the fall standard scores in time 1 and the spring standard scores for time 2, these results indicate that children improved their math skills above and beyond what was expected in a span of five months (Figure 2A). There was no main effect of attendance (beta = 0.015, *SE* = 0.017, *t*(61.81) = 0.90, *p* = 0.370) or interaction with attendance (beta < 0.001, *SE* = 0.012, *t*(47) = 0.02, *p* = 0.984), suggesting that these learning gains were independent of participating in the intervention program. All told, children’s average scores shifted from low average (M = 81.02, SD = 10.07) to almost average (M = 88.12, SD = 12.17).

**Positive Attitude toward Math.** There was no main effect of time, nor attendance, nor interaction (*p* > 0.320), indicating that children’s attitudes toward math remained stable from time 1 (M = 4.12, SD = 0.78) to time 2 (M = 4.22, SD = 0.75) regardless of their participation in the intervention program (Figure 2B).

**Inhibitory control**. Analyses of accuracy in the H&F task yielded a significant effect of incongruent block (beta = −0.864, *SE* = 0.215, *t*(235) = 4.02, *p* < 0.001) and mixed block (beta = −1.424, *SE* = 0.215, *t*(235) = 6.63, *p* < 0.001), indicating that children’s performance was modulated by the block difficulty, with congruent, the easiest, followed by incongruent and then mixed (Figure 2C). There was also a marginal interaction between time and incongruent block (beta = 0.567, *SE* = 0.304, *t*(235) = 1.87, *p* = 0.063), suggesting that there was a marginal moderate increase in children’s performance in the incongruent block from time 1 to time 2. Notably, these improvements were not modulated by attendance to the intervention, with no main effect or interactions with attendance (all *p*s > 0.35).

### Intervention Results

In the following sections, to provide evidence on the specificity of our results and rule-out test-re-test effects, we performed three linear mixed models, one for each format. We included the standardized accuracy scores (z-scores) as the dependent variable and added time (T1 vs. T2), counting (consistent and misleading) as within-participant fixed factors, and attendance as a between-participants factor, as well as the interactions, with participant as random intercept. Then, we calculated the marginal means, which were estimated from the linear mixed model, of the significant effects and interactions and performed pairwise comparisons of these estimated means to determine T1 and T2 differences across different number of completed sessions.

#### Continuous Proportional Reasoning

**FIGURE 3:**
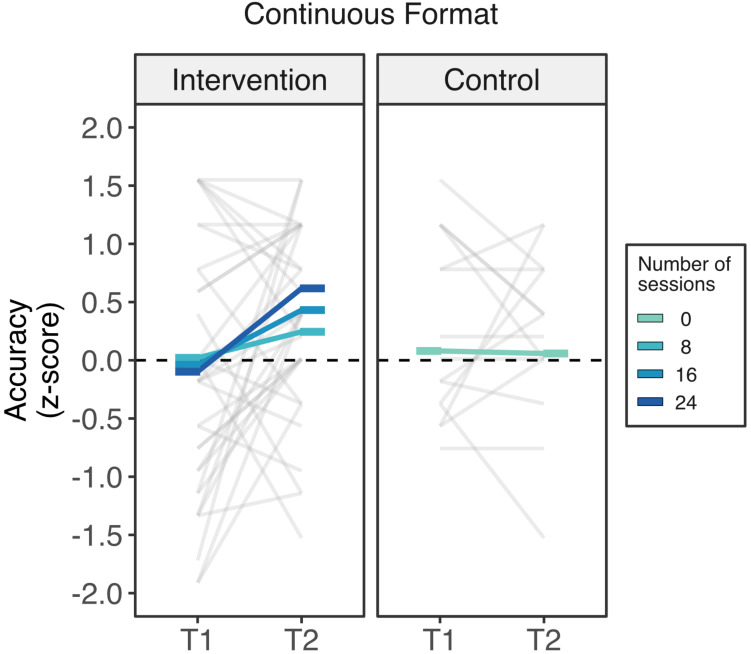
Intervention outcomes in the non-symbolic continuous format of the Spinners task by the number of completed sessions. Children who attended all the sessions of the Cuisenaire intervention showed a reliable increase in their ability to compare spinners presented in the continuous format while children were part of the control group showed exhibited no improvement. Rods represents the estimated marginal means across different number of sessions of attendance from 0 sessions (control group) to 24 sessions (darkest blue).

While there were no improvements in the control group, children in the intervention group improved in line with the number or sessions attended (Figure 3). The linear mixed model revealed a marginal interaction between time and attendance (beta = 0.033, *SE* = 0.198, *t*(141) = 1.70, *p* = 0.091), confirming that children’s improvements scaled with their attendance. To further characterize this interaction, we looked at the T1 and T2 marginalized mean differences at (0, 8, 16, and 24 sessions). These T1 vs. T2 comparisons indicated that while children who participated in zero sessions did not increase their performance (mean difference = 0.02, *SE* = 0.20, 95% CI [−0.415, 0.372], *p* = 0.913), children who received 8 (mean difference = 0.224, *SE* = 0.138, 95% CI [−0.046, 0.494], *p* = 0.107) and 16 sessions (mean difference = 0.470, *SE* = 0.151, 95% CI [0.174, 0.766], *p* = 0.0023) showed small increases. Finally, according to the model, children who completed the full intervention (24 sessions) had a large increment in their performance (mean difference = 0.716, *SE* = 0.227, 95% [0.271, 1.161], *p* = 0.002).

#### Outcomes in Discrete Proportional Reasoning

**FIGURE 4:**
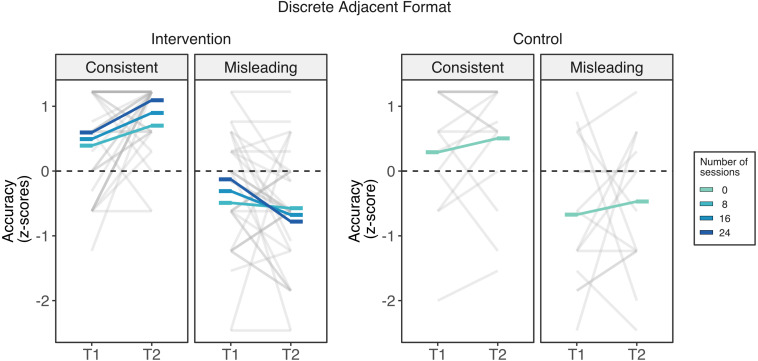
Intervention outcomes in the non-symbolic discrete adjacent format of the Spinners task across groups. Unexpectedly, although improvements in the ability to compare pairs of spinners whose number of segments matched the proportional information varied across did not vary by the number of completed session, children’s ability to compare spinners when the number of segments was misleading was negatively related to the number of sessions. That is, children who completed 24 session (darkest blue line) showed the largest losses. Rods represents the estimated marginal means across different number of sessions of attendance from 0 sessions (control group) to 24 sessions (darkest blue).

*Discrete adjacent* format. Children in the intervention group showed a decrease in their performance in the discrete adjacent misleading trials the more they attended to the intervention (Figure 4). This pattern of results was confirmed by the linear mixed model. This analysis yielded a negative linear effect of counting (beta = −0.963, *SE* = 0.251, *t*(141) = 3.84, *p* < 0.001), which was qualified by a marginal three-way interaction between counting, time, and attendance (beta = −0.047, *SE* = 0.025, *t*(141) = 1.92, *p* = 0.057). We performed two follow-up linear mixed models, one for each counting condition, with attendance and time as fixed factors and participants as random slopes. While the linear mixed model for the counting consistent condition did not yield any significant effect or interaction (*p* > 0.21), there was a marginal interaction between time and attendance in the misleading condition (beta = −0.036, *SE* = 0.018, *t*(47) = 1.94, *p* = 0.058). Unexpectedly, this interaction indicated that children who attended more sessions decreased their performance in the misleading trials. The posthoc pairwise comparisons indicated that children who completed the full 24 sessions would be expected to have a significant decrease (beta = −0.652, *SE* = 0.298, 95% CI [−1.236, −0.068], *p* = 0.034), but not for those attending 16 or fewer sessions. In summary, these results indicate that the intervention might have increased the use of effective strategies for comparing discrete consistent trials but impair performance for cases in which there is a conflict between whole-number and proportional information.

**FIGURE 5:**
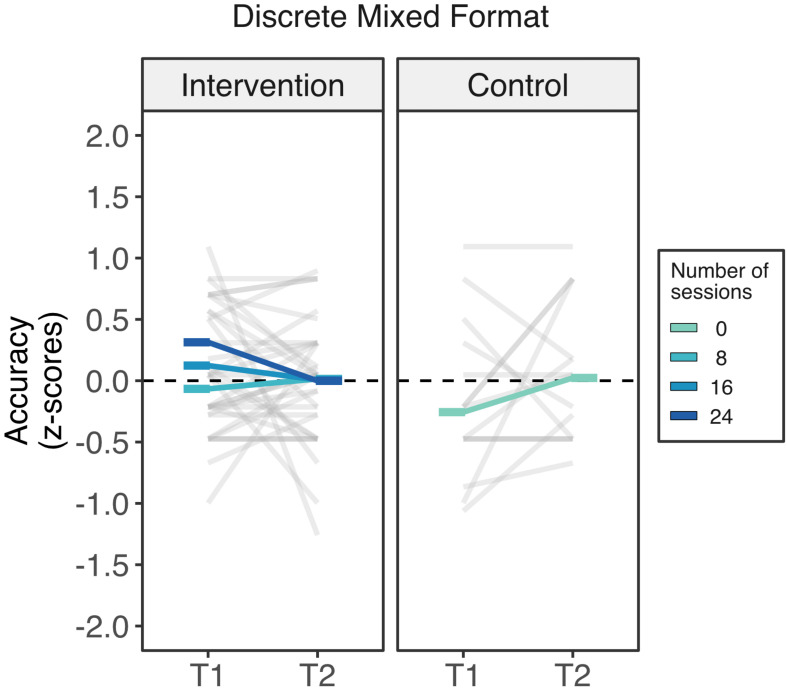
Intervention outcomes in the non-symbolic discrete mixed format of the Spinners task across groups. Changes in the discrete mixed format from time 1 to time 2 did not vary by attendance to the intervention. Rods represents the estimated marginal means across different number of sessions of attendance from 0 sessions (control group) to 24 sessions (darkest blue).

*Discrete mixed* format. There were no meaningful changes in either of the two groups in the discrete mixed format (Figure 5). Consistently, the linear mixed model yielded a negative linear effect of counting (beta = −1.176, *SE* = 0.245, *t*(141) = 4.80, *p* < 0.001), confirming the expected effect of lower performance on counting misleading problems. There was neither effect of time (*p* = 0.135) nor any two- nor three-way interactions involving time and attendance (*p* > 0.20).

### Exploratory Analyses: The Relationship Between Academic, Attitudinal, and Cognitive Measures and Changes in Performance

**FIGURE 6:**
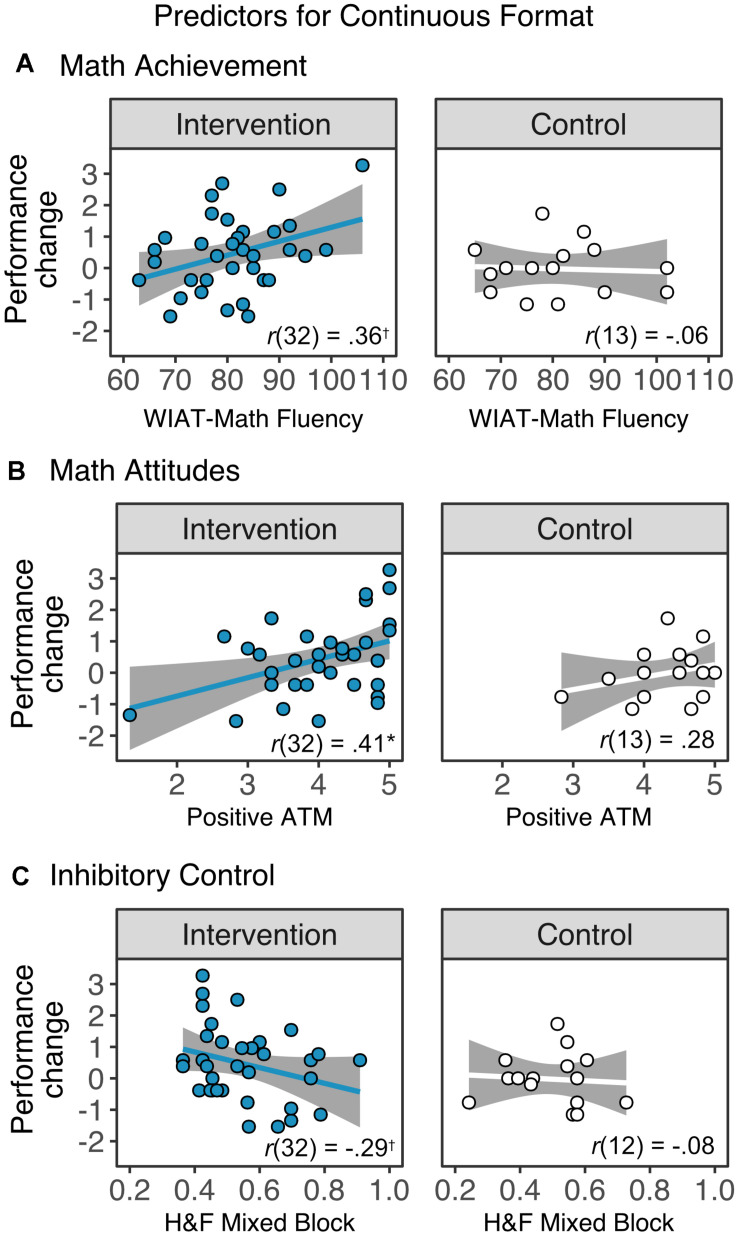
Associations between changes in performance in the continuous format of the Spinners task and **(A)** math achievement, as measured by the fluency subtests from the Wechsler Individual Achievement Test (WIAT)-III, **(B)** attitudes toward math (ATM), and **(C)** inhibitory control, as measured by the mixed block of the Hearts and Flowers (H&F) task. Children in the intervention group with better math skills and more positive attitudes toward math showed marginally larger learning gains. In contrast, children with lower inhibitory control skills in this group showed marginal larger gains. Notably, none of the correlations with change scores in the control group reached significance. *p_*corr*_ < 0.05, **^†^***p*_*corr*_ < 0.10.

**FIGURE 7:**
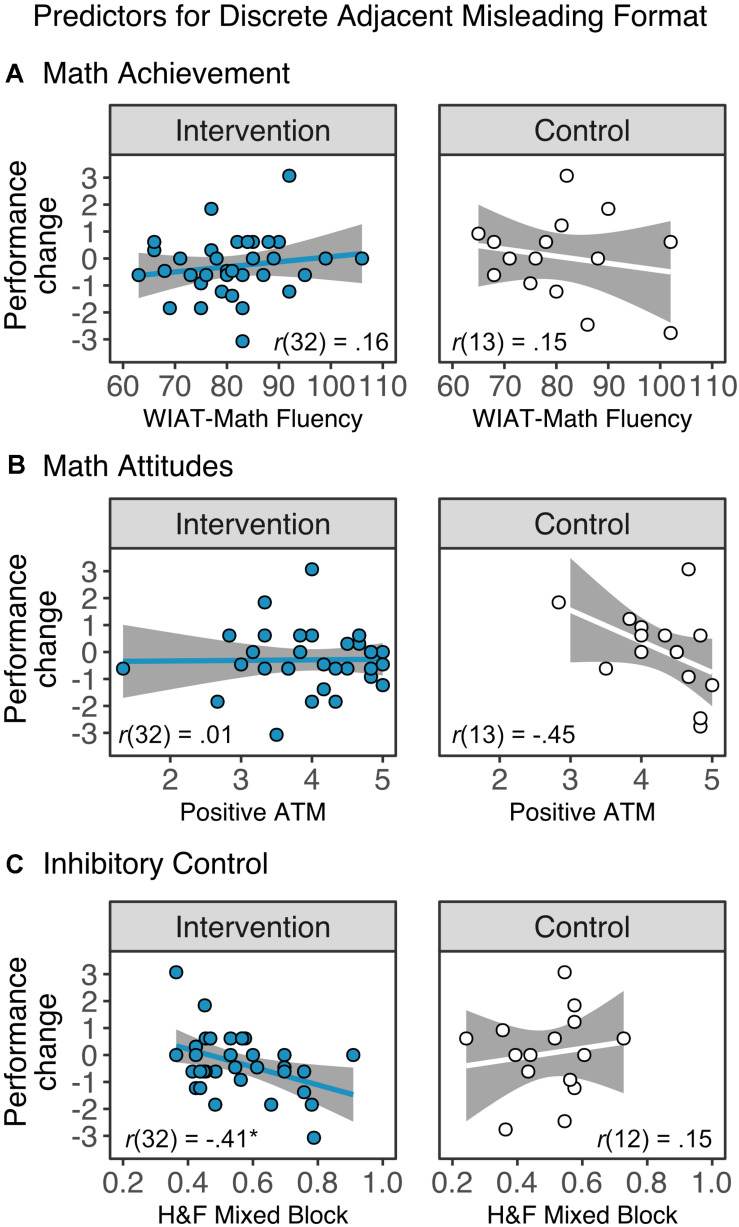
Associations between changes in performance in discrete adjacent misleading trials of the Spinners task and **(A)** math achievement, as measured by the fluency subtests from the Wechsler Individual Achievement Test (WIAT)-III, **(B)** attitudes toward math (ATM), and **(C)** inhibitory control, as measured by the Hearts and Flowers (H&F) task. There were no significant correlations between individual difference measures and changes in the discrete adjacent misleading trials, except for a negative association between the initial inhibitory control of the intervention group and learning gains. *p_*corr*_ < 0.05.

To explore whether changes in proportional reasoning skills of children in the intervention group related to children’s initial academic, cognitive and motivational skills, we performed Pearson correlations between the changes in the spinners task (T2 – T1) in continuous (Figure 6) and discrete adjacent (Figure 7) format and children’s initial arithmetic fluency skills, attitudes toward math, and inhibitory control ability. We also examined the relationship between the developmental changes in proportional reasoning and academic, cognitive, and motivational skills in the control group. Importantly, we looked at these relationships separately as these predictor measures did not relate to each other (absolute *r*-values < 0.15, *p*s > 0.32). Finally, in cases where significant associations were found in the intervention group but not the control group, we formally tested for interactions using a linear regression analyses with the learning gains in proportional reasoning as the dependent variable, and the interaction between number of sessions and the significant predictor (math performance, attitudes toward math, or inhibitory control) as the moderator term.

#### Predictor Effects in Continuous Proportional Reasoning

After correcting for multiple comparisons using false discovery rate correction (Benjamini and Hochberg, 1995), there was a marginal association between the learning gains in the continuous format and T1 math scores (*r*(32) = 0.36, *p*_*corr*_ = 0.057) of children in the intervention group; that is, children with better math skills during the initial evaluation showed larger learning gains. We also found a significant positive relationship between attitudes toward math and children’s learning gains (*r*(32) = 0.41, *p*_*corr*_ = 0.046), showing that children who had more positive attitudes toward math showed larger gains. Conversely, there was a marginal negative relationship between the time 1 score in the mixed block of the H&F task and the learning gains in non-symbolic continuous proportional reasoning skills (*r*(32) = −0.29, *p_*corr*_* = 0.095). This pattern of results suggests that our intervention program resulted in larger gains for children with low-inhibitory control skills. In contrast, none of the correlations with change scores in the control group (all *p*_*corr*_’s > 0.83).

A linear regression, with math performance as a moderator showed a significant interaction between math and attendance (beta = 0.0007, SE = 0.0003, *t* = 2.08, *p* = 0.043), suggesting that children who attended more sessions and had better math performance showed larger gains. There was also a marginal interaction between attendance and inhibitory control (beta = −0.046, SE = 0.027, *t* = 1.69, *p* = 0.097), suggesting that children with low inhibitory control but a higher number of completed sessions benefitted the most from the intervention. Finally, there was no interaction between attendance and attitude toward math scores (*p* = 0.29).

#### Predictor Effects in Discrete Proportional Reasoning

There was a negative correlation for discrete proportional reasoning for misleading trials. Children in the intervention group with lower inhibitory control had larger gains in misleading trials (*r*(32) = −0.41, *p*_*corr*_ = 0.045). There were no significant correlations between individual difference measures and changes in the discrete adjacent misleading trials in the control group (all *p*_*corr*_’s > 0.27).

The linear regression to predict learning gains in the discrete adjacent misleading condition with attendance and inhibitory control as independent variables did not yield a significant interaction between these two factors (*p* = 0.34).

## Discussion

Training studies have the potential of uncovering the mechanisms that underpin effective learning programs (Rosenberg-Lee, 2018). Emerging research has pointed to non-symbolic proportional reasoning as a building block for later fraction understanding (Siegler et al., 2011; Matthews et al., 2016). In the current study, we examined the malleability of this skill by looking at the effects of a 24-session proportional reasoning intervention (Powell, 2019), using Cuisenaire rods, on second-grade students’ ability to compare non-symbolic continuous and discrete ratios. We found that children who went through essentially the complete intervention showed a large increase in their ability to compare non-symbolic continuous proportions in a different representational format, annulus-shaped figures. However, we observed a decline in their ability to compare discretized proportions, specifically, when the absolute number of pieces contradicted the proportional information. In contrast, while children who did not attend any sessions did not show any improvement or decline in their proportional reasoning skills, even after the five months, children who received at least 16 sessions showed a small but consistent increment in their continuous proportional skills. Finally, we found a positive predictor role of children’s aptitudes and attitudes in mathematics on the continuous learning outcomes, but a negative role for children’s inhibitory control skills.

### Cuisenaire Rod Intervention Improves Non-symbolic Continuous Proportional Reasoning

Children who completed the 24 sessions of our intervention showed an increase in their non-symbolic continuous proportional reasoning. These results are consistent with previous findings showing that non-symbolic proportional reasoning is malleable through training non-symbolic estimation skills (Gouet et al., 2020) and mapping between non-symbolic and symbolic formats (Wortha et al., 2020). Importantly, in contrast to previous interventions implemented in highly structured, individual, computerized environments, the current intervention took place in an ecologically valid context (small group instruction in the children’s classroom) and used inexpensive materials (Cuisenaire rods). Further, our approach was implemented slowly over five months compared to only a few days in prior work (e.g., Gouet et al., 2020). These features contribute to the ecological validity of our intervention, which could facilitate its implementation by teachers. Conversely, these features may complicate implementing this approach in a large scale, randomized control trial needed to establish efficacy.

Our study also expands these previous findings by showing that children can transfer their gains in proportional reasoning to a different non-symbolic representation after an intervention focused explicitly on conceptual thinking. In the current study, children extended their recently acquired understanding of proportional reasoning with one model (lines) to one in which they did not receive any practice (annulus-shaped figures). Significantly, these results cannot be attributed to test-retest effects as children who did not attend any session did not show any improvement.

During the intervention, children viewed proportions represented in both non-symbolic and symbolic formats. Working with these two formats might have facilitated transferring proportional reasoning skills from one modality (rods) to another (annulus-shaped figures). Children compared the relative lengths of rods and used verbal and written symbolic expressions to represent them. Thus, children might have developed representations of proportions that were not constrained by the perceptual properties of the stimuli they were manipulating. Consistent with this interpretation, previous fraction interventions have shown that using different non-symbolic representations of proportions (e.g., circles and tiles) helps children transfer gains in fraction comparison ability to fraction magnitude estimation skills in children with low working memory (Fuchs et al., 2014). Future studies should examine whether the non-symbolic or the symbolic features of the intervention or the combination of both, drove children’s improvements in proportional reasoning.

### Discrete Proportional Reasoning Is Hindered by the Intervention

In contrast to the relative ease of processing continuous proportions, discrete quantities pose a significant challenge to non-symbolic proportional reasoning. Students not only have to manipulate the proportional quantities but also have to override the whole-number information (Jeong et al., 2007; Boyer et al., 2008; Abreu-Mendoza et al., 2020). Two lines of research suggest that changes in continuous proportional reasoning should also lead to improvements in discrete reasoning. First, there is an emergent body of research suggesting that priming non-symbolic continuous proportions leads to short-term improvements in processing discrete quantities (Boyer and Levine, 2015; Hurst and Cordes, 2018; Abreu-Mendoza et al., 2020). Second, a recent proposal has suggested a cognitive system devoted to processing proportional quantities regardless of format and modality (Matthews et al., 2016). Therefore, we hypothesized that by training proportional reasoning with a particular focus on a continuous magnitude (the relative lengths of Cuisenaire rods), children would not only improve their ability to compare continuous proportions but also discrete ones. However, contrary to this hypothesis, children who completed the 24 sessions of the intervention showed a decline in their ability to compare discrete quantities, specifically in contexts where whole-number information interfered with the proportional one. These results beg the question if proportions are processed in a modality-independent manner (Matthews et al., 2016; Park et al., 2020), why do gains in continuous proportional reasoning not transfer to discrete quantities? We offer two potential explanations: one related to our intervention’s instructional structure and the other to the developmental trajectory of discrete proportional reasoning.

One feature of our intervention that may have hindered children’s discrete reasoning skills is the nature of the Phase 1 activities. Some of these activities involved combining two or more rods and contrasting them with rods of larger lengths. Although these activities focused on a continuous magnitude, length, children still might have focused on discrete elements (rods). Moreover, comparing proportions in the context of whole-number interference was not an explicit topic of the intervention. This omission may have been compounded by features of the intervention aimed at increasing student agency: participants proposed the proportion problems they would work on during the sessions; thus, students may have never encountered counting misleading problems (Rosenberg-Lee, 2021). These two features of the intervention combined with the on-going instruction of whole-number operations and units that children received during the second grade (Common Core State Standards Initiative, 2020a) might have led children to focus on the absolute number of pieces instead of the proportional information in our discrete outcome task.

The protracted development of discrete proportional reasoning might have also played a part in children’s decrease in this ability. For instance, a recent study found that even fifth-graders with low-fraction knowledge performed at chance level in a match-to-sample task that involved discrete-adjacent stimuli (Begolli et al., 2020). Only children with at least a moderate level of fraction knowledge could manipulate discrete quantities, suggesting that *symbolic* proportional skills might be required to overcome the misleading discrete information while manipulating non-symbolic proportional quantities (Begolli et al., 2020). The authors suggest that children might need more symbolic proportional experience to disregard discrete information successfully.

In summary, consistent with these previous studies, our current findings suggest that to help children in the protracted development of discrete proportional reasoning (Jeong et al., 2007), children may require direct instruction that brings to conscious attention the interference of whole-number knowledge to proportional reasoning (Rosenberg-Lee, 2021), explicitly linking continuous and discrete proportional reasoning and a more extensive period of symbolic proportional instruction.

### Math Achievement and Inhibitory Control Moderate Learning Outcomes

The third goal of this study was to examine the predictive relations of measures of math aptitude, math attitude, and inhibitory control abilities with changes in performance in non-symbolic continuous and discrete proportional reasoning skills. Despite our small sample size, but consistent with longitudinal studies of fractions (Jordan et al., 2013) and general math growth (Dowker et al., 2019), both initial math performance and attitudes toward math showed a positive relationship with children’s learning outcomes in the intervention group. Notably, only math performance had a positive interaction with attendance when predicting learning gains in continuous proportional reasoning, suggesting that children with higher math skills and that attended more of the intervention showed the greatest gains in their ability to compare proportions of continuous quantities.

Conversely, children’s initial inhibitory control skills in the intervention group were negatively related to their learning gains in continuous proportional reasoning and losses in discrete proportional reasoning. Further, inhibitory control had a negative interaction with attendance in predicting gains in the continuous format. That is, children with low inhibitory control but with high attendance benefit the most from the intervention. This pattern of results might appear counterintuitive at first sight, especially in light of previous studies reporting a positive relationship between inhibitory control and non-symbolic (Abreu-Mendoza et al., 2020) and symbolic proportional processing (Gomez et al., 2015; Avgerinou and Tolmie, 2019; Coulanges et al., 2021). However, a parsimonious interpretation of these results is that the current intervention is beneficial to remediate the proportional reasoning skills of those students with low inhibitory control skills. Indeed, a previous study examining aptitude-treatment interactions in the context of fraction learning reported a similar result: a conceptually rich program was more effective to improve fraction knowledge of children with low working memory, than a fraction fluency intervention (Fuchs et al., 2014). However, the current study leaves unanswered whether this effect is specific to the assessed executive function (i.e., inhibitory control). Future studies contrasting different non-symbolic proportional reasoning programs that include a more comprehensive cognitive assessment would provide insights on these questions. Further, it raises the possibility that exposing children with poor inhibitory control to proportional stimuli may help them to build up non-symbolic representation that could be helpful for fraction processing (Matthews et al., 2016; Kalra et al., 2020). Relating changes in proportional reasoning to improvements in symbolic fraction understanding, especially among learners with inhibitory control deficits, is vital in understanding the mechanisms supporting this remediation approach (Rosenberg-Lee, 2018).

### Considerations and Future Directions

While recent interventions on non-symbolic and symbolic fraction knowledge have involved individual repetition of computerized tasks (Fazio et al., 2016; Gouet et al., 2020), the current intervention program comprised tasks that involved children working in small groups, using well-known educational materials, Cuisenaire rods. This ecologically valid implementation allowed children to build a conceptual understanding of proportions by first working with whole non-symbolic and symbolic magnitudes (first phase) and slowly transition to non-symbolic and symbolic proportions (second phase). However, the intervention’s cumulative nature, which required students to attend all sessions, make children’s irregular and unsystematic attendance a significant limitation of the study. For example, while some children completed the eight sessions of the first phase but did not attend any of the second phase session, others attended 15 out of the 16 sessions of the second phase but none of the first one, making it difficult to disentangle the effects of the number of sessions from to content of the session. Our results indicated that children who attended the complete intervention showed the largest changes in their performance; however, only six out of the 34 children of the intervention group attended the complete intervention (24 sessions), and only 14 children attended 80% or more sessions. This low rate in completing the intervention might have contributed to the marginal significance of some of the results. Larger samples are needed to verify pattern of results especially gains in continuous but losses in discrete misleading. While short-term computerized instruction can produce gains (Fazio et al., 2016; Gouet et al., 2020), conceptual instruction has been shown to have long-standing positive impacts (Misquitta, 2011). Future research should contrast the cost-benefits of long-term, group-based, conceptual instruction and short-term, individual interventions on children’s proportional reasoning.

## Conclusion

The current study examined the effects of a proportional reasoning intervention, in which children transition from non-symbolic to symbolic fraction comparisons and expressions, on children’s ability to compare ratios of continuous and discrete quantities. Our results showed that children who completed the intervention increased their ability to compare non-symbolic continuous proportions. However, contrary to our expectations, children decreased their ability to compare misleading discretized proportions. These results provide further evidence on the malleability of non-symbolic continuous proportional reasoning and speak to the persistence of difficulties with discrete proportional skills.

## Data Availability Statement

The datasets presented in this article are not readily available because participants did not consent to data sharing. Requests to access the datasets should be directed to MR-L, miriam.rosenberglee@rutgers.edu.

## Ethics Statement

The studies involving human participants were reviewed and approved by Rutgers University Institutional Review Board. Written informed consent to participate in this study was provided by the participants’ legal guardian/next of kin.

## Author Contributions

RA-M, KA, AP, and MR-L conceived the study. RA-M and LC collected the data. RA-M performed the analyses. RA-M, AP, and MR-L wrote the original draft. RA-M, LC, AP, and MR-L reviewed and edited the manuscript. All authors contributed to the article and approved the submitted version.

## Conflict of Interest

The authors declare that the research was conducted in the absence of any commercial or financial relationships that could be construed as a potential conflict of interest.
